# On Cod-Liver Oil in Phthisis

**Published:** 1852-08

**Authors:** 


From the London Journal of Medicine, Dec., 1851, p. 1132.

*On Cod-Liver Oil in Phthisis.* By Dr. Walshe.

The conclusions at which Dr. Walshe has arrived, with regard to the use of cod-liver oil in the treatment of phthisis, are as follows:

1. That it more rapidly and effectually induces improvement in the general and local symptoms than any other known agent. 2. That its power of *curing* the disease is undetermined;—I mean here, by 'curing' the disease, its power of causing, along with suspension of progress, such change in the organism generally, as shall render the lungs less prone to subsequent outbreaks of tubercles, than after suspension occurring under other agencies. 3. That the mean amount of permanency of the good effects of the oil is undetermined. 4. That it relatively produces more marked effects in the third, than in the previous stages. Opinions the most diverse have been held on this point; M. Taufflied taught that it had little or no effect on phthisis, if at all advanced; M. Pereyra *reduced the size of cavities in a few weeks* by its administration. 5. That it increases weight in favorable cases with singular speed, and out of all proportion with the actual quantity taken;—and hence it must in some unknown way save waste, and render food more readily assimilable. 6. That it sometimes fails to increase weight. 7. That in the great majority of cases, whe e it fails to increase weight, it does little good in other ways. 8. That it does not relieve dyspnœa out of proportion with other symptoms. 9. That the effects traceable to the oil in the most favorable cases are increase of weight, suspension of colliquative sweats, improved appetite, diminished cough and expectoration, cessation of sickness with cough, and gradual disappearance of active physical signs. 10. That in some cases it cannot be taken, either because it disagrees with the stomach, impairing the appetite (without itself obviously nourishing), and causing nausea, or because it produces diarrhœa. 11. That in the former case it may be made palatable by association with a mineral acid; and in the latter prevented from affecting the bowels by combinations with astringents. 12. That intra-thoracic inflammations and hæmoptysis are contra-indications to its use, but only temporarily so. I have repeatedly given the oil within a day or two of the cessation of hæmoptysis, without any return taking place. 13. Diarrhœa, if depending on chronic peritonitis, or secretive change, or small ulcerations in the ileum, is no contra-indication to the use of the oil; even the profuse diarrhœa caused by extensive ulceration of the large bowel is not made worse by it. 14. That the good effects of the oil are *cæteris paribus* directly as the youth of those using it,—a singular fact, and which probably may one day (when the textural peculiarities of youth and age are better understood) aid in giving a clue to its mode of action.

